# Applying Evidence-Based Medicine in Telehealth: An Interactive Pattern Recognition Approximation

**DOI:** 10.3390/ijerph10115671

**Published:** 2013-10-31

**Authors:** Carlos Fernández-Llatas, Teresa Meneu, Vicente Traver, José-Miguel Benedi

**Affiliations:** 1 Instituto Universitario de Investigación de Aplicaciones de las Tecnologías de la Información y de las Comunicaciones Avanzadas (ITACA), Universitat Politècnica de València, Camino de Vera S/N, Valencia 46022, Spain; E-Mails: tmeneu@itaca.upv.es (T.M.); vtraver@itaca.upv.es (V.T.); 2 Instituto Tecnológico de Informática (ITI), Universitat Politècnica de Vaència, Camino de Vera S/N, Valencia 46022, Spain; E-Mail: jbenedi@dsic.upv.es

**Keywords:** evidence based medicine, interactive pattern recognition, personalized medicine, clinical guidelines, probabilistic model

## Abstract

Born in the early nineteen nineties, evidence-based medicine (EBM) is a paradigm intended to promote the integration of biomedical evidence into the physicians daily practice. This paradigm requires the continuous study of diseases to provide the best scientific knowledge for supporting physicians in their diagnosis and treatments in a close way. Within this paradigm, usually, health experts create and publish clinical guidelines, which provide holistic guidance for the care for a certain disease. The creation of these clinical guidelines requires hard iterative processes in which each iteration supposes scientific progress in the knowledge of the disease. To perform this guidance through telehealth, the use of formal clinical guidelines will allow the building of care processes that can be interpreted and executed directly by computers. In addition, the formalization of clinical guidelines allows for the possibility to build automatic methods, using pattern recognition techniques, to estimate the proper models, as well as the mathematical models for optimizing the iterative cycle for the continuous improvement of the guidelines. However, to ensure the efficiency of the system, it is necessary to build a probabilistic model of the problem. In this paper, an interactive pattern recognition approach to support professionals in evidence-based medicine is formalized.

## 1. Introduction

With the arrival of the Internet, the globalization of health and the increasing of new opportunities for improving the care process by sharing knowledge, the paradigm of how physicians should face their daily work needs to be restated. Currently, not only is the number of patients that search for information about their illness on the Internet growing [1,2], but also, even junior physicians are starting to base their diagnosis and treatment decisions on the information gathered on the Internet [3]. In this way, the use of the Internet for disseminating health-related knowledge in a more complete and effective way is now becoming a reality. This is one of the aims of the telehealth paradigm.

The idea of telehealth is not new. Since the nineteen nineties, the classical paradigm of clinical practice has been continuously in discussion. More formally, Nikelson defines telehealth as *the use of telecommunications to provide health information and care across distance* [4]. Telehealth philosophy has redesigned the framework of how physicians should face their daily work. The increase in the variability of patients that physicians can virtually visit, the possible lack of direct contact and the quantity of information available in a continuous care paradigm cause a profound change essential in the classical physicians daily practice. The classical paradigm in which the physician is considered an isolated element that trusts in their own experience to diagnose and apply adequate treatments to a patient is now changing to another that makes use of well-known scientific knowledge as the basis to provide better and more effective treatments. These facts are forcing medical doctors to adapt their daily practice with new methods and technologies, moving from experience to evidence-based medicine (EBM) [5] to address this problem.

EBM promotes the integration of the best biomedical evidence to physicians’ daily clinical practice. EBM requires that physicians are active and continuously complement their expertise with the information available in big libraries of clinical cases. With the arrival of the digital era, the possibility to find information about illness diagnosis and treatment on the Internet is exponentially increasing. Thanks to the current rapid and ubiquitous Internet access, it is possible to access incredibly large digital libraries over the Internet. That opportunity can be exploited by physicians, allowing them to apply very recent scientific medical studies to their current patients in very little time after their publication. In the case of telehealth, where the physician may not have direct access to the patient, the use of patient-centered protocols to monitor and empower the patient in their own care process is critical. For that, the standardization of care continuity and the use of preventive patient-centered protocols will provide an efficient and effective way to profit from the penetration of technology. In other words, the use of care protocols for standardizing health may be the solution to allow holistic control of the patient integrated with the daily practice of the general practitioner. In fact, EBM and clinical guidelines have been used for creating specific telehealth protocols [6].

However, although EBM aims to be patient centered, taking into account the patient’s choices in the process of care [7], there is a growing skepticism in the way EBM and clinical guidelines have been deployed in a personal health approach [8,9]. Clinical guidelines are continuously improved by the results achieved in clinical trials. Clinical trials are based on stratification and segmentation, but not on individualized patients. In this way, clinical guideline critics argue that the characteristics of clinical trial population inclusion criteria differ critically from individual patients, which should be the target of guidelines [8]. For the telehealth paradigm, the problem is even worse. The classical statistical approach of clinical trials is based on general probabilistic models that analyze the effect of treatments or diagnosis methods in different groups looking for evidence that demonstrates the validity of the processes. However, these probabilistic models do not take into account characteristics, such as the dynamic change of the patient’s history or the iterative effect of physicians decisions on patient behavior, depending on the patient’s personality. In our vision, this information is critical in a telecare process. Continuous control of disease involves the patient and the physician in a very coupled, dynamic and iterative flow in which the decisions of physicians and the responses of patients seem to be as important as the biomedical data gathered in the care process. For that, to be able to construct patient-centered clinical guidelines in a holistic way, the creation of probabilistic models that reflect the statistical dependencies and correlations among the variables in the care protocol of a disease is necessary, taking into account not only patient characteristics, but also the effect of general practitioner decisions. The use of that probabilistic model within the use of clinical trial statistical methods will enable the maximizing of the efficiency and accuracy in each optimization iteration of the clinical guideline.

In this paper, an interactive pattern recognition probabilistic approach based on EBM principles is formalized. This approach takes into account the whole care process, as well as the relationship among the stakeholders involved. This paper is organized as follows. First, EBM and clinical guideline concepts are defined in more detail. Secondly, our EBM probabilistic model is presented, and finally, a short discussion about the results concludes the paper.

## 2. Evidence-Based Medicine Principles and Clinical Guidelines

According to Sacket et al. [7], evidence-based medicine *is the conscientious, explicit and judicious use of the current best evidence in making decisions about the care of individual patients. The practice of evidence-based medicine means integrating individual clinical expertise with the available external clinical evidence from systematic research.*

In EBM, the clinical competence of individual physicians is integrated with the best clinical evidence available through systematic research [10]. In this way, EBM is aimed at physicians making their diagnosis decisions and treatments based on the most updated biomedical literature by making a critical argument and taking into account their personal experience. EBM promotes the creation of clinical guidelines and protocols to guide clinical decisions. Those protocols and guidelines should be integrated into the professionals’ daily practice. However those protocols are not intended to be strictly followed, but to empower physicians to achieve cost-effective and high quality care paths. In summary, EBM promotes:

- *Intensive use of the biomedical literature*: The integration of biomedical literature with daily practice will allow the decisions of physicians to be based on statistical evidence. To allow that integration, it is necessary that this information be accessible to physicians in an easy and practical way.
- *Critical reading of the literature based on personal experience*: Due to the high variability of human behavior and multi-pathological patients, it is very usual that patients that have the same illness have different responses to the same treatment. Therefore, the evidence taken from the biomedical literature should be used only as a valid complement to the personal experience of the physician.
- *Patient-centered care*: The EBM advocates for patient involvement in the care process. The empowerment of patients and informal caregivers not only will allow for a more effective self-care of patients, but also allows for better understanding of their illness, allowing them to prevent disease complications.

The application of evidence-based medicine principles requires the continuous analysis of the literature and of clinical cases to support the physicians’ daily practice. To achieve such empowerment, the first step is to provide physicians with current biomedical knowledge at their work environment. One of the tools used by EBM to disseminate scientific evidence to the medical community is clinical guidelines. Clinical guidelines are documents whose objectives are to support physicians’ clinical decisions by providing them with scientifically validated evidence to diagnose, manage and treat each specific illness. More formally, in [11], a clinical guideline is defined as being *systematically developed statements to assist practitioners and patient decisions about appropriate healthcare for specific circumstances*. A more recent definition was presented in [12] as *statements that include recommendations intended to optimize patient care that are informed by a systematic review of evidence and an assessment of the benefits and harms of alternative care options.*

Clinical guidelines identify, summarize and evaluate medical knowledge based on scientific evidence. Those documents suppose a continuously updated state-of-the-art prevention, diagnosis, prognosis and treatments that currently have demonstrated evidence of their effectiveness on specific illnesses. Those clinical guidelines are becoming reference documents for health professionals, supporting them in their daily decisions. Clinical guidelines have demonstrated their advantages [13,14], supporting health professionals in the continuous improvement of clinical outcomes, reducing the variability in clinical practice, forcing experts to unify criteria and providing greater cost effectiveness in daily practice.

The use of information and communication technologies (ICT) can be a way to disseminate clinical guidelines. In this line, there are different digital libraries that make clinical guidelines available over the Internet, like PUBMED [15], Fisterra [16] or Cochrane [17]. These repositories have indexed a high quantity of clinical guidelines, available for use by physicians and medical teams.

These documents are exponentially increasing in number and are continuously updated by biomedical researchers. This continuous improvement requires an iterative process that is currently being discussed [18,19,20,21]. To allow for the correct deployment of the EBM, it is necessary that the most recent and contrasted scientific evidence be reflected in the clinical guidelines. In this iterative process, the scientific discoveries are used by medical expert communities for updating existing clinical guidelines, providing better hypotheses for caring for patients and becoming, step by step, the perfect protocols that cover all the issues for an illness.

To maximize the efficiency of this iterative process, the creation of a probabilistic model allows us to work in a formal framework that ensures the theoretical correctness of our hypothesis and, then, to obtain better results in practice. There are some works that point to the Bayesian theories as the most accurate formal framework to approach the achievement of biomedical evidence [22], and some also advising about the problems of working with other widely used validation methods, like *p*-value [23]. In those papers, the authors show how the Bayesian theory can help in the validation of the evidence achieved in biomedical research, but they do not take into account one of the fundamentals of EBM: daily practice integration. Our hypothesis is that incorporating daily practice into the probabilistic model will allow us to achieve a better understanding of the EBM dependencies and, then, get better results for the improvement of clinical guidelines.

In this paper, we propose a Bayesian approximation to the whole process of EBM integrating biomedical research with the general practitioners daily practice.

## 3. Evidence-Based Medicine in the Interactive Pattern Recognition Framework

The number of existing clinical pathways and guidelines available on the Internet for use by physicians and medical teams is exponentially increasing and continuously improving. However, the great amount of information available makes it practically impossible for physicians to be properly updated. The pattern recognition (PR) paradigm [24] can be a solution for supporting physicians in their daily practice. PR provides a formal framework that allows for the development of mechanisms for supervision and inference of the most accurate protocols. Additionally, the PR framework also allows us to design new adaptation techniques based on personal profiles.

Interacting with machines has proven to help many human activities. However, machines can also take advantage of human feedback to improve their performance. In this context, the new interactive pattern recognition (IPR) framework has been recently proposed [25]. This proposal enables interaction between a human and a PR system, allowing the system to learn from this interaction, as well as adapting the system itself to the human behavior. IPR has been applied in different PR fields. These include interactive transcription of handwritten and spoken documents, computer-assisted language translation and interactive text generation and parsing, among others [25]. In this section, we aim to apply the principles of IPR to the management of evidence-based medicine (EBM).

The EBM-based guidelines are adapted depending on the specific characteristics of current patients. In an IPR scenario, these adaptions can be the basis for the automatic inference of new guidelines, helping in the continuous improvement of them by using the pattern recognition approach. In other words, the application of IPR to EBM will allow us to iteratively adapt the clinical guidelines to the specific features of patients, as well as to automatically improve the new guidelines using the information of each individual adaption. In addition, these inferred guidelines are expressed in a formal way. On the one hand, the formalization of clinical guidelines allows for the possibility of defining models that represent, adequately and without ambiguities, the clinical care process. On the other hand, it also enables building automatic methods to estimate the formal guidelines, as well as mathematical models for optimizing the iterative cycle for the continuous improvement of guidelines.

### 3.1. Interactive Pattern Recognition Framework

In order to allow for an effective application of the pattern recognition paradigm, it is important to analyze the recognition problem from a probabilistic perspective. In the classical PR paradigm, we can formulate the problem as: Let *x* be an input stimulus, observation or signal and *y* a hypothesis or output, which the system has to derive from *x*. Let *M* be a *model* or a set of models used by the system to derive its hypotheses. In general, *M* is obtained through an automatic *batch* training process from a given *training corpus* of the task being considered.

The idea of the classic PR paradigm is to find the output hypothesis, *ŷ*, that maximizes the posterior probability, *Pr*(*y*|*x*), of the hypothesis, *y*, when the entry data, *x*, is produced. Using a model, *M*, this is approximated as:

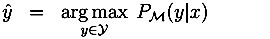
(1)

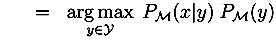
(2)
where *Y* is the (possibly infinite) set of valid output hypotheses. In many cases, it is difficult to estimate *P_M_*(*y*|*x*) (Equation (1)), and it is better to apply the Bayes rule to achieve the decomposition of Equation (2). The term, *P_M_*(*x*), has been dropped, since it does not depend on the maximization variable, *y*.

The terms in Equation (2) are the *likelihood* model, *P_M_*(*x*|*y*), that represents the relationship between the input stimulus and its output hypothesis and the, *prior* *P_M_*(*h*), that represents the well-formedness of the output hypothesis.

### 3.2. IPR Approach for EBM

However, the application of the the classical pattern recognition framework to EBM is not realistic, as it is enunciated. This is because the inferred models rarely are perfect, and the inference method cannot be fail-safe. For that, the presence of a health professional who ensures the validity of the hypothesis inferred by PR systems is needed. In order to do that, there are two possible approaches for incorporating the health expert into the process of inference:

- *Post-process approach*: The PR system offers a solution, and the expert analyzes and adapts it.
- *Interactive approach*: The expert is involved in the IPR building process of the solution.

**Figure 1:**
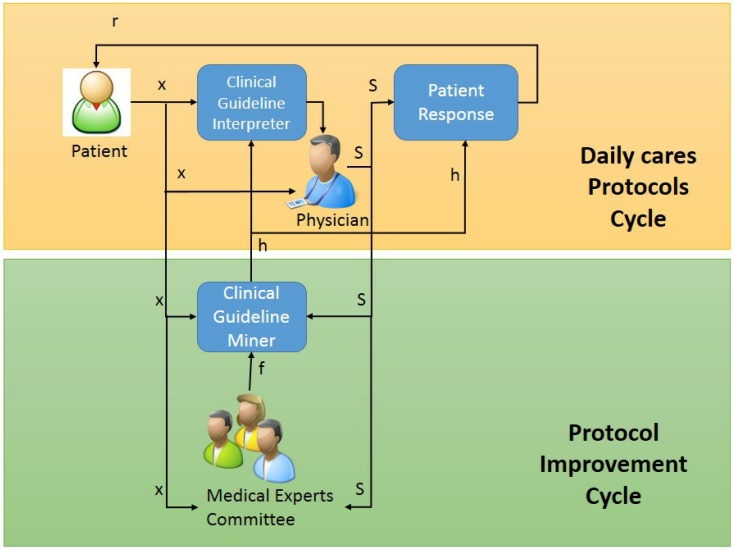
Proposed dual interactive system.

In this paper, we present a new IPR approach for supporting EBM in the formalization of clinical guidelines and their optimization and adaption to daily care. That means that health professionals are continuously involved in the process of identification, adaption and optimization of the clinical guideline hypothesis. In Figure 1, a graphical description of the presented model is shown. According to the EBM philosophy, we have separated the problem into two different stages: the daily care protocol cycle and the interactive protocol improvement cycle.

#### 3.2.1. Daily Care Protocol Cycle

The daily care protocol cycle represents the usual path followed by the patient involved in a care process following a clinical guideline. In this cycle, the patient is in touch with his physician. Depending on the more adequate clinical guideline, *h*, and the multiple signs and symptoms of the patient, *x*, a different status, *s*, is sug/gested that is associated with the appropriate treatment or diagnostic method. A patient can respond differently to the treatment depending on his pathologies or personal characteristics, which can affect the treatment results (for example, adherence). These results will become new entries, *r*, to the next cycle iteration. This model can be seen as a classical dialogue system, where the treatment is the response to the signs and symptoms of the patient. In each iteration of the daily process, the physician analyzes the data, *x*, the status, *s*, and the current clinical guideline, *h*, to correct the status of the patient within the clinical guidelines. If considered necessary, the physician is able to modify the patient status. That implies the application of a different treatment or diagnostic method that has not been directly suggested by the clinical guidelines. For example, if according to the data gathered the current hypothesis (clinical guideline) is that the patient severity is high, but the physician considers that it is not accurate, he can change the status to low severity, applying the treatments proposed for this case by the clinical guidelines.

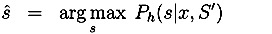
(3)

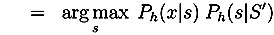
(4)

In Equation (3) *h* is the clinical guideline associated with the patient, *S′* is the history of all the previous states visited by the patient in the clinical guideline defined as *h*, supervised and modified, if needed, by physicians in each iteration. This equation represents that the system obtains the best status, *ŝ*, that has been associated with the best treatment, using all the information gathered (*x*, *h*, *S′*).

Using Bayes and applying the restriction that *P_h_*(*x*|*s*, *S′*) does not depend on *S′*, we can reach Equation (4). This assumption, similar to the application in other well-known probabilistic models, like Hidden Markov Models (HMM), allows us to reduce the problem, making it easier to solve. Observing Equation (4), it is important to note that *P_h_*(*s*|*S′*) is the *a priori* probability of *s* being compatible with *S′*; so, we take into account only the *s* compatible with the current hypothesis, *h*, and the history, *S′*. Each status has associated treatments and diagnostic methods that cause a response, *r*, in the patient. This response will be used in the next care iteration as new gathered data, *x*.

In this line, the physician can correct the status in each iteration by selecting different treatments and diagnostic methods. However, according to the model, the physician is not able to change the structure of the hypothesis, meaning that the physician cannot change the clinical guideline. If, according to the physician’s experience, the patient needs a different treatment not included in the hypothesis, then the patient should go out of the clinical guideline, starting a classical process of care.

#### 3.2.2. Interactive Protocol Improvement Cycle

As we have seen in the previous section, the physician is not able to modify the structure of the current clinical guideline, *h*. The improvement of the clinical guidelines takes place in the interactive protocol improvement cycle. In this stage, a group of independent health experts is involved in the interactive learning process to offer new and optimized clinical guidelines to physicians for daily practice.

The treatments and diagnostic methods used by physicians in daily care, as well as the responses of the patients can be used to infer new clinical guidelines, better adapted and optimized for daily practice using interactive pattern recognition methodologies. The aim of this section is to build the probabilistic formal framework of IPR to support experts in this second cycle.

The second cycle exposed in Figure 1 represents the continuous improvement of the clinical guidelines. In this stage, the patient’s signs and symptoms, *x*, and the diagnostics methods and treatments, *s*, will be used to infer a new improved clinical guideline, *h*. In addition, this continuous improvement also depends on the medical expert committee decisions, *f*, which apply human knowledge to the clinical guideline, as well as the previous clinical guideline used, *H*, due to the close relationship between the treatment followed and the entry data. For this model, we assume that new advances and scientific evidence are included in the *f* function and are filtered and applied according to the medical expert committee decisions.

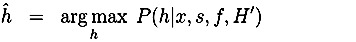
(5)

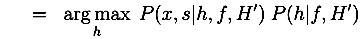
(6)

In the Equation (5), *h* is the clinical guideline, *H′* is the history of applied clinical guidelines, *x* is the data collected from the patient, *s* is the status of the patient corrected by the physician and *f* is the feedback of the expert group that is able to modify the structure of the guideline by inserting, deleting and modifying the status available. Intuitively, the new clinical guideline depends on the information gathered from daily care (*x*, *s*), the expert committee decisions, *f*, and the history of the previous hypothesis, *H′*.

Applying Bayes in a similar way to the previous section, we can achieve Equation (6), where (*x*, *s*) are the entry samples and *P* (*x*, *s*|*h*, *f*, *H′*) can be simplified by making a naive Bayes’ assumption: the input observation, *x*, and the current state in the process, *s*, are statistically independent variables, given *h*, *f* and *H′*, obtaining:

*P*(*x*, *s*|*h*, *f*, *H′*) = *P*(*x*|*h*, *f*, *H′*) *P*(*s*|*h*, *f*, *H′*)

Simplifying dependencies on medical expert committee feedback, *f*, and the historical hypothesis, *H′*, we can write the prediction of *ĥ* in more detail:

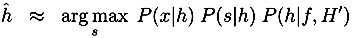
(7)


According to this equation, in order to maximize clinical guidelines improvement, it is not only necessary to take into account the concordance of the new clinical guideline with the signs and symptoms of the patient, (*P* (*x*|*h*)), but also, the concordance with the treatments followed, (*P* (*s*|*h*)). This is because the selection of the correct treatments and diagnostic methods is related to the response of the patient gathered in the form of signs and symptoms. In addition, we need to take into account (*P* (*h*|*f*, *H′*)), which is the probability of the hypothesis, *h*, that is compatible with the expert decisions, *f*, and the history of the guidelines, *H′*.

## 4. Discussion and Conclusion

In this paper, a double cyclic interactive paradigm for applying pattern recognition to EBM is formalized. In the first cycle, the daily care of the patient is formalized by the clinical guideline and supervised by the experience of the physician in the interaction with the patient. In the second cycle, the clinical guidelines, used by physicians in daily practice, are constructed and optimized based on clinical evidence from the results achieved in studies made by biomedical researchers.

According to probability theory, the systems that are intended to build good clinical guidelines should maximize the probability of the acceptance of data gathered from patient *x*, the probability of the acceptance of the physician interactions *S* and the *a priori* probability of the model. That means that the treatments, diagnostic methods and other patient/physician interactions are as important as the data gathered (biomedical, demographic, *etc.*) from the patient. Then, all these interactions directly affect the success of a clinical guideline and should be taken into account, together with the rest of the data in clinical cases, to improve the clinical guidelines.

Intuitively, daily practice physician interactions can provide information related to the experienced *feelings* of the physician or to the behavior of the patient facing the treatment (*i.e.*, adherence), which can be critical in the selection of the disease protocol and can be very difficult measure directly from the patient. The application of a treatment in itself can be a diagnostic method or even voided treatments can provide information (*i.e.*, placebo effect). For that and according to our theoretical results, the treatments, diagnostic methods and other patient/physicians interactions that are applied should be added to statistical datasets, to learn more and to be adapted to reality and accurate models.

Furthermore, the deductive decisions provided by the medical expert committee in order to improve the clinical guideline based on previous hypothesis are decisive. Therefore, the creation of systems and models that allow for these experts to be more aware of the physicians’ daily practice will produce more effective and accurate clinical guidelines.

In order to provide a framework to evaluate the proposed system, we advocate for one based on usability and quality of service. As IPR is a supervised model, the error expected in each iteration is zero. This is because the experts correct the errors in each iteration to ensure a safe deployment of the clinical guidelines in real cases. In that case, indicators, such as the number of iterations needed to achieve a complete (or acceptable) clinical guideline, the number of corrections made by physicians and medical experts in each iteration, the quality of service or the satisfaction of the users (not only physicians, but also patients), can be used to evaluate the system. However, this evaluation should be made in real cases with real patients in order to evaluate the impact of this model in daily practice.

To take advantage of this paradigm, we need to use a formal language to represent the hypothesis. This formal representation can be interpreted by computers, and then, care can be deployed using the telehealth paradigm. Those protocols can be supervised by physicians using the daily care cycle interactive pattern formalized in this paper. In the second interactive cycle, we suggest the creation of interactive data mining processes that incorporate the patient and professional. This not only will take into account the results of classical statistical approaches, but also the interaction among professionals and patients, like dynamic treatments and patient decisions, like adherence, which will be integrated in the model, being more accurate and adapted to reality. However, to allow for a fully interactive system, we need representation models that are easy to understand by human experts. This is because, the easier a language is to be understood, the easier the hypothesis is to be supervised and optimized.

In this way, to apply the formalism achieved, we suggest the use of finite state-based workflows as the hypothesis language, as well as process mining algorithms, to infer the new hypothesis. Finite state-based workflows are designed to be easily understood and have been used to represent guidelines [26,27]. Finite state systems can be automatized by computers and can be propagated through telehealth applications, allowing for the supervision of physicians in daily care. Process mining algorithms [21] can be used to infer workflows that can be supervised, optimized and corrected by health experts to achieve better formal clinical guidelines in the next iteration of the process.

## References

[1] Kummervold P.E., Chronaki C.E., Lausen B., Prokosch H.U., Rasmussen J., Santana S., Staniszewski A., Wangberg S.C. (2008). eHealth trends in Europe 2005–2007: A population-based survey. J. Med. Int. Res..

[2] Powell J., Inglis N., Ronnie J., Large S. (2011). The characteristics and motivations of online health information seekers: Cross-sectional survey and qualitative interview study. J. Med. Int. Res..

[3] Hughes B., Joshi I., Lemonde H., Wareham J. (2009). Junior physician's use of Web 2.0 for information seeking and medical education: A qualitative study. Int. J. Med. Inf..

[4] Nickelson D.W. (1998). Telehealth and the evolving health care system: Strategic opportunities for professional psychology. Prof. Psychol. Res. Pract..

[5] Straus S.E., Richardson W.S., Glasziou P., Haynes R.B. (2005). Evidence-Based Medicine: How to Practice and Teach EBM.

[6] Britton B.P. (2003). First home telehealth clinical guidelines developed by the American Telemedicine Association. Home Healthc. Nurse.

[7] Sackett D.L., Rosenberg W.M.C., Gray M.J.A., Haynes B.R., Richardson S.W. (1996). Evidence based medicine: What it is and what it isn't. BMJ.

[8] Goldberger J.J., Buxton A.E. (2013). Personalized medicine vs. guideline-based medicine. JAMA J. Am. Med. Assoc..

[9] Romana H.W. (2006). Is evidence-based medicine patient-centered and is patient-centered care evidence-based?. Health Serv. Res..

[10] Elstein A.S. (2004). On the origins and development of evidence-based medicine and medical decision making. Springer.

[11] Field M., Lohr K. (1990). Clinical Practice Guidelines: Directions for a New Program.

[12] Graham R., Mancher M., Wolman D.M., Greenfield S., Steinberg E. (2011). Clinical Practice Guidelines We Can Trust.

[13] Grol R., Grimshaw J. (2003). From best evidence to best practice: Effective implementation of change in patients' care. Lancet.

[14] Cosby J.L. (2006). Improving patient care: The implementation of change in clinical practice. Qual. Saf. Health Care.

[15] PubMed Library National Library of Medicine and The National Institutes of Health PubMed Library. http://www.pubmed.gov.

[16] Fisterra. http://www.fisterra.com/fisterrae/.

[17] The Cochrane Collaboration COCHRANE Library. http://www.cochrane.org/index.htm.

[18] Eden J., Wheatley B., McNeil B., Sox H. (2008). Knowing What Works in Health Care: A Roadmap for the Nation.

[19] Buchan H.A., Currie K.C., Lourey E.J., Duggan G.R. (2010). Australian clinical practice guidelines a national study. Med. J. Aust..

[20] Shaneyfelt TM C.R. (2009). Reassessment of clinical practice guidelines: Go gently into that good night. JAMA.

[21] Fernandez-Llatas C., Meneu T., Benedi J.M., Traver V. Activity-Based Process Mining for Clinical Pathways Computer Aided Design. Proceedings of the IEEE Engineering in the 32nd Annual International Conference of the Medicine and Biology Society.

[22] Ashby D., Smith A.F. (2000). Evidence-based medicine as Bayesian decision-making. Stat. Med..

[23] Goodman S.N. (1999). Toward evidence-based medical statistics 2: The bayes factor. Ann. Intern. Med..

[24] Duda R.O., Hart P.E., Stork D.G. (2001). Pattern Classification.

[25] Toselli A.H., Vidal E., Casacuberta F. (2011). Multimodal Interactive Pattern Recognition and Applications.

[26] Sedlmayr M., Rose T., Rhrig R., Meister M. A Workflow Approach towards GLIF Execution. Proceedings of the European Conference on Artificial Intelligence (ECAI).

[27] Fernandez-Llatas C., Pileggi S.F., Traver V., Benedi J.M. Timed Parallel Automaton: A Mathematical Tool for Defining Highly Expressive Formal Workflows. Proceedings of the IEEE 2011 Fifth Asia Modelling Symposium AMS.

